# Elective genomic screening: results of the implementation of a whole genome sequencing program at a medical check-up unit in Spain

**DOI:** 10.3389/fgene.2025.1722462

**Published:** 2026-01-23

**Authors:** Bibiana Palao, Miriam Leon-Otegui, Raquel Bernad, Maria Moreno-Coca, Elena Ordoñez, Elena Góngora, Isabel Castilla, Miguel Sogbe, Oscar Beloqui, Ana Patiño-García, Vincenzo Cirigliano, Luis Izquierdo

**Affiliations:** 1 Veritas Intercontinental, Madrid, Spain; 2 Veritas Intercontinental, Barcelona, Spain; 3 Department of Internal Medicine, Clínica Universidad de Navarra, Pamplona, Spain; 4 Department of Internal Medicine, Check-up Unit, Clínica Universidad de Navarra, Pamplona and Madrid, Spain; 5 Department of Medical Genomics, Clínica Universidad de Navarra, Pamplona, Spain

**Keywords:** elective genome, genomic check-up, genomic screening, preventive genomics, preventive medicine, genome sequencing

## Abstract

Elective Genomic Testing (EGT) can identify individuals at risk for actionable conditions that would not come to clinical attention following current testing guidelines. We describe the results of a checkup unit from a leading Spanish University hospital (Clínica Universidad de Navarra, Spain) that has incorporated EGT to their regular clinical practice. Medical anamnesis, biochemistry, low-intensity whole body scan and EGT with interpretation of over 560 genes related to actionable adult-onset diseases (Veritas Intercontinental, Spain) was performed in 400 participants, including medical consultation before and after the checkup. Clinically relevant variants were identified in 79/400 participants (19.8%). Thirteen individuals (3.3%) presented with clinical variants included in the American College of Medical Genetics and Genomics secondary finding list (ACMG SF list); 69.2% of these variants showed potential association with personal or family history (PFH). The study presents the results of the first hospital integrating EGT into the checkup unit.

## 1 Introduction

Population screening for disease-causing genetic variants can identify individuals at risk for preventable conditions such as hereditary cancer, heart disease, and other disorders, who would otherwise be missed by current clinical testing guidelines. Genome sequencing was implemented in the clinical practice as an effective approach for the diagnosis of rare genetic disorders (Bick et al., 2019). However, the utility of genome sequencing is not limited to patients with a medical indication, generally healthy individuals may also benefit from this testing approach, which is referred to as Elective Genomic Testing (EGT).

EGT is a natural progression of well-established screening practices, such as newborn screening (Dubay and Zach, 2023; Wilcken and Wiley, 2008), carrier screening (Chokoshvili et al., 2018; Gregg et al., 2021; Sparks, 2020), or the ACMG SF list reporting (Miller et al., 2023). The difference between diagnostic genome and EGT is the phenotype-driven variant analysis. Concerning the possible genetic readouts, diagnostic genome primary findings include variants that may explain an aspect of the patient’s PFH. Variants that do not appear to be associated with PFH at the time of testing are considered as secondary findings, but are nevertheless clinically relevant.

There is growing evidence endorsing the potential of genomic screening to prevent or reduce the impact of actionable conditions in patients’ health (Savatt et al., 2025; Foss et al., 2022; Alarcon Garavito et al., 2023; Jones et al., 2025). Testing individuals with PFH suggestive of genetic origin is an effective strategy but may exclude many at-risk patients. For conditions such as cancer and heart disease, identifying patients at risk facilitates risk management and early detection (Buchanan et al., 2020).

There are three conditions considered by the CDC (Centers for Disease Control and Prevention, United States) as Tier 1 genomic applications, based on strong evidence supporting early detection and intervention: hereditary breast and ovarian cancer syndrome, Lynch syndrome, and familial hypercholesterolemia. Genomic testing for these conditions is considered to reduce morbidity and mortality for millions of people (Khoury et al., 2022) since around 1%–2% of the population are carriers of a pathogenic (P) or likely pathogenic (LP) variant related to CDC Tier 1 genes (Abul-Husn et al., 2021). Around 75% (Grzymski et al., 2020) of individuals presenting genetic variants do not meet criteria to be tested based on PFH and thus are unaware of carrying a genetic variant that increases their risk for those specific conditions.

Screening unselected cohorts for hereditary breast and ovarian cancer syndrome (*BRCA1* and *BRCA2* genes) yields similar outcomes, facilitating the identification of individuals harboring relevant variants, and 50% of these individuals will not have a PFH indicating their increased cancer risk (Manickam et al., 2018; Gabai-Kapara et al., 2014; Abul-Husn et al., 2019).

Published evidence indicates that whole genome sequencing screening in a generally healthy population reveals genetic variants associated with disease risk in approximately 17%–19% of individuals (Hou et al., 2020; Cochran et al., 2021), frequently unsuspected prior to testing. A recently published study shows that testing for the 81 genes in ACMG SF list v3.2 identifies 1 out of 30 individuals with a genetic variant known to increase the risk of disease (Savatt et al., 2025), many of them associated with a reduced life span (Jensson et al., 2020). EGT could be used not only for screening adult-onset conditions, but also for analyzing other well-described clinically relevant variants, given the availability of comprehensive genomic information. An innovative application of EGT is the calculation of polygenic risk score (PRS) for common diseases, such as sporadic breast cancer, atrial fibrillation, coronary artery disease or type II diabetes (Khera et al., 2018).

The published evidence regarding population elective genomic screening is growing (Savatt et al., 2025; Foss et al., 2022; Alarcon Garavito et al., 2023; Jones et al., 2025), but still limited. The available studies have been performed using whole exome sequencing or targeted panels for specific conditions, showing highly variable and difficult-to-compare findings. Furthermore, there are also discrepancies in the scope of the different programs and publications. The scope varies from genes related to CDC Tier1 conditions (Grzymski et al., 2020), genes described in different versions of the ACMG SF gene list (Jensson et al., 2020), whole genome sequencing (Hou et al., 2020; Jones et al., 2025) including sometimes and as an add carrier status, pharmacogenomic information and traits or ancestry (Foss et al., 2022). This lack of clinical consensus in the genomic screening strategy implies a challenge to compare different testing approaches but enriches the genomic landscape to nurture an appropriate environment that allows the development of an optimal and equitable global genomic screening initiative. The only converging conclusion of the different publications is that elective genomic screening is a cornerstone of preventive medicine and should be considered for implementation in the clinical setting to some extent.

In this article, we provide the results of the first hospital checkup unit from a leading Spanish University Hospital offering EGT for actionable adult-onset diseases as a standard of care, together with biochemical and image testing.

## 2 Materials and methods

### 2.1 Study cohort

The retrospective study cohort consists of 400 unselected adults who attended the Clínica Universidad de Navarra Medical Check-up Unit (Madrid and Pamplona facilities, Spain) to participate in the Genomic Check-up service. All participants read and signed the informed consent. The participants were adults, with a mean age 51.32 (SD 11.20, range 19–80 years), being 59.5% of them males and 40.5% females. In our cohort, 30.8% of participants (123/400) had a positive personal history of cancer, liver disease, diabetes, cardiovascular disease and/or inflammatory conditions. On the other hand, approximately 53% of participants (211/400) had a positive family history (first and/or second degree relative/s) of cancer, and approximately 11.8% (47/400) had a positive family history of cardiovascular disease. A total of 31 patients presented with family history of both cancer and cardiovascular diseases.

### 2.2 Genomic check-up

The Genomic Check-up service consists of a complete medical anamnesis, comprehensive biochemistry, low-intensity whole body scan and EGT (myGenome, Veritas Intercontinental, Spain) which includes the patient’s whole genome sequencing and its subsequent interpretation. The service includes a pre and post-test genetic counselling consultation. All the genomic findings were interpreted in the context of the patient´s complete clinical information to establish future medical management.

Pre-test genetic consultation was scheduled to inform about potential outcomes and findings, benefits, and limitations of current knowledge and technology, together with possible implications of the results for other family members. Post-test genetic consultation was also arranged to deliver the results, explain the implications, answer any questions and establish if any additional clinical testing or adapted medical management were needed.

### 2.3 Whole genome sequencing

For the EGT, blood samples collected at Clínica Universidad de Navarra were sent and processed at Veritas laboratory. Extracted genomic DNA was processed with the DNA PCR-Free Prep Tagmentation® (Illumina Inc., San Diego, United States of America), libraries sequenced 2 × 150 bp reads on NovaSeq 6,000 next-generation sequencers (Illumina Inc., San Diego, United States of America) using S4 flow cells. Sequencing average raw output was 100 Gb per sample to obtain an average coverage of 30X on the covered region of approximately 4.5 billion bases including coordinates described as highly reliable by Zook et al. (2016) and a selection of markers of ancestry and genetic traits.

WGS data analysis was performed with a custom proprietary bioinformatics pipeline (Phoenix v1.3.3, Veritas Intercontinental, Spain), which uses both Bayesian and Heuristic-based statistical variant callers to filter variants. After BCL conversion FASTQs were aligned to hg19 (build 37.1) human reference genome using BWA (version 0.7.5a) (Li, 2013), resulting data sorted and indexed using Samtools (version 1.9.0) (Li et al., 2009) and duplicates marked using Picard tools (version 2.3.0) (https://broadinstitute.github.io/picard/). Two variant callers were used (Freebayes 0.9.21-24 [(Garrison and Marth, 2012)] and DeepVariant v0.7.0 [(Poplin et al., 2018)]) with the resulting VCFs merged (VCF tools v 0.1.11 [(Danecek et al., 2011)]) before annotation using Variant Effect Predictor v86 (McLaren et al., 2016) and SnpEff v4.3t (Cingolani et al., 2012).

Initial filtering is based on population frequency, variant type, and variant classifications in the ClinVar and HGMD databases, together with loss of function variants in a subset of genes and pre-established variants related to risk alleles, traits and pharmacogenomics. Only inherited germline variants are detected and interpreted.

The pharmacogenomic pipeline analyze the pre-established variants that affect drug efficacy, dose adjustment, and adverse effects for certain drugs. For genes that use the nomenclature of haplotypes in the form of “star alleles,” the pipeline calls the variants that define the different star alleles, and the haplotype is assigned based on the detected genotype with a proprietary design. The haplotype *1 is usually assigned by default if none of the analyzed variants are found. Cyrius (version 1.1) (Illumina Inc) is used to call CYP2D6 haplotypes. Ancestry determination is performed using a model-based clustering method based on multilocus genotype data to infer population structure and assign individuals to populations (Pritchard et al., 2000).

### 2.4 Genomic data analysis

It is necessary to limit the scope of the EGT to actionable adult-onset diseases for which screening methods or early detection are defined. To this end, the ACMG SF list v3.2 (Miller et al., 2023) served as the primary driver for disease selection, expanding to additional genes with available scientific evidence related to the conditions listed, or to other variants with available clinical management such as FV Leiden (Laryea and Champagne, 2013) or pharmacogenomic variants.

The bioinformatics platform enables the genomic analysis to integrate in a single assay all the relevant genetic information clinically useful in a healthy setting. The variants detected were classified in different categories i) monogenic actionable conditions, ii) carrier status, iii) pharmacogenomics, iv) risk alleles, and v) ancestry. The pipeline also provides the automated analysis of a section of traits, which, despite having no clinical impact, could potentially be interesting for nutrition or exercise adaptation purposes.

For Mendelian disorders, variant interpretation was restricted to a subset of 560 genes related to clinically actionable disorders as well as genes associated with recessive conditions. Variants filtered by the pipeline went through deep curation according to ACMG guidelines (Richards et al., 2015). Variants classified as P or LP were reported. Benign variants, likely benign variants, and variants of uncertain significance (VUS) were not reported as they are not considered to be clinically useful for healthy individuals.

A separate interpretation framework was applied to variants that are associated with an increased risk but are generally not expected to cause disease on their own, such as risk alleles. The risk alleles included in the test were selected based on quality of phenotyping, statistical significance, and replication of results (Senol-Cosar et al., 2019). Pharmacogenetic data analysis and interpretation are based on a subset of guidelines from the Clinical Pharmacogenetics Implementation Consortium (CPIC), the PharmGKB resource (currently renamed into ClinPGx https://www.clinpgx.org/including also CPIC information), and the guidelines from the Dutch Pharmacogenomics Working group, and in some cases, governmental regulatory agencies such as the European Medicines Agency and the United States of America Food and Drug Administration.

## 3 Results

### 3.1 Identification of clinically relevant variants in actionable genes

The algorithm used for ancestry analysis, indicated that 91.0% (364/400) of the participants were primarily European, while 3.3% (13/400) were Hispanic, 0.3% (1/400) African, 0.3% (1/400) Asian, 0.3% (1/400) Ashkenazi, and 5% (20/400) presented mixed ancestry.

Of all the variants detected in the 400 patients, a total of 90 were classified as clinically relevant, and were included in the final report (Table 1). The 90 variants corresponded to 34 unique variants distributed in 29 genes, 12 of those variants were present in more than one patient. Of these 34 variants, 30 (88.2%) were previously described and 4 (11.8%) were novel loss-of-function variants (NLoF) that were detected by the pipeline but were absent from the clinical literature or genomic databases. Interestingly, one of these NLoF was identified in two patients.

**TABLE 1:** Clinically relevant variants identified in the 400 patients.

Gene	Condition	Variant	Section	Variant type	Category	ACMG SF 3.2	Variant occurrence
*APC*	Colorectal cancer	*APC* c.3920T>A (p.Ile1307Lys), NM_000038, HET, ER	Clinical	PD	Cancer	YES	2
*APOE* ^*f*^	Hyperlipoproteinemia type III	*APOE* c.460C>A (p.Arg154Ser), NM_000041, HET, P	Clinical	PD	Cardiovascular	NO	1
*ATM*	Cancer susceptibility related to *ATM* variants	*ATM* c.329_330delGA (p.Arg110Lysfs*4), NM_000051, HET, LP	Clinical	NLoF	Cancer	NO	1
*ATM* ^*c*^	Cancer susceptibility related to *ATM* variants	*ATM* c.5188C>T (p.Arg1730*), NM_000051, HET, LP	Clinical	PD	Cancer	NO	1
*BARD1* ^*d*^	Cancer susceptibility related to *BARD1* variants	*BARD1* c.1728_1731dup (p.Ser578Alafs*11), NM_000465, HET, LP	Clinical	NLoF	Cancer	NO	1
*BRCA1* ^*g*^	Hereditary breast and ovarian cancer syndrome	*BRCA1* c.5152 + 2T>G (p.?), NM_007294, HET, LP	Clinical	PD	Cancer	YES	1
*BRCA2*	Hereditary breast and ovarian cancer syndrome	*BRCA2* c.2808_2811del (p.Ala938Profs*21), NM_000059, HET, P	Clinical	PD	Cancer	YES	2
*BRIP1*	Cancer susceptibility related to *BRIP1*	*BRIP1* c.1702_1703delAA (p.Asn568Trpfs*9), NM_032043, HET, P	Clinical	PD	Cancer	NO	1
*CHEK2*	Cancer susceptibility related to *CHEK2*	*CHEK2* c.1100delC (p.Thr367Metfs*15), NM_007194, HET, P	Clinical	PD	Cancer	NO	1
*CHEK2* ^*a*^	Cancer susceptibility related to *CHEK2*	*CHEK2* c.319 + 2T>A, NM_007194, HET, LP	Clinical	PD	Cancer	NO	1
*CTLA4*	Autoimmune lymphoproliferative syndrome (ALPS)	*CTLA4* c.160G>A (p.Ala54Thr), NM_005214, HET, VUS with solid evidence	Clinical	PD	Cancer	NO	2
*DSG2* ^*b*^	Arrhythmogenic right ventricular cardiomyopathy (MAVD)	*DSG2* c.146G>A (p.Arg49His), NM_001943, HET, P	Clinical	PD	Cardiac	YES	1
*ELN*	ELN-related disorders	*ELN* c.1748-1G>C, NM_000501, HET, LP	Clinical	NLoF	Cardiac	NO	2
*ENG*	Hereditary haemorrhagic telangiectasia	*ENG* c.1272 + 2T>C, NM_000118, HET, LP	Clinical	PD	Organ health	YES	1
*F8*	Haemophilia A	*F8* c.396A>C (p.Glu132Asp), NM_000132, HEM, LP	Clinical	PD	Clotting	NO	1
*FLNC*	Filaminopathies	*FLNC* c.5205_5206del (p.Cys1735*), NM_001458, HET, LP	Clinical	NLoF	Cardiac	YES	1
*G6PD*	Glucose-6-phosphate dehydrogenase deficiency (X-linked)	*G6PD* c.292G>A (p.Val98Met), NM_000402, HEM, P	Clinical	PD	Endocrine and metabolic	NO	1
*G6PD* ^*e*^	Glucose-6-phosphate dehydrogenase deficiency (X-linked)	*G6PD* c.292G>A (p.Val98Met), NM_000402, HET, P	Clinical	PD	Endocrine and metabolic	NO	2
*HFE*	Susceptibility to hereditary haemochromatosis	*HFE* c.187C>G (|)845G>A (p.His63Asp(|)Cys282Tyr), NM_000410, COMP HET, ER	Clinical	PD	Organ health	NO	3
*HFE* ^*b,c,g*^	Susceptibility to hereditary haemochromatosis	*HFE* c.845G>A (p.Cys282Tyr), NM_000410, HET, ER	Clinical	PD	Organ health	NO	18
*KCNH2*	Long QT syndrome	*KCNH2* c.2863C>G (p.Leu955Val), NM_000238, HET, VUS with solid evidence	Clinical	PD	Cardiac	YES	1
*MITF* ^*f*^	Cutaneous melanoma	*MITF* c.1255G>A (p.Glu419Lys), NM_198159, HET, ER	Clinical	PD	Cancer	NO	2
*PALB2*	*PALB2*-predisposition to cancer	*PALB2* c.1240C>T (p.Arg414*), NM_024675, HET, P	Clinical	PD	Cancer	YES	1
*PROC*	Protein C deficiency	*PROC* c.1212dup (p.Pro405Alafs*20), NM_000312, HET, P	Clinical	PD	Clotting	NO	1
*RAD51C* ^*d*^	Ovarian cancer	*RAD51C* c.404G>A (p.Cys135Tyr), NM_058216, HET, LP	Clinical	PD	Cancer	NO	1
*RET*	Multiple endocrine neoplasia	*RET* c.2410G>A (p.Val804Met), NM_020975, HET, P	Clinical	PD	Cancer	YES	1
*RTEL1*	*RTEL1*-related disorders	*RTEL1* c.2956C>T (p.Arg986*), NM_016434, HET, P	Clinical	PD	Cancer	NO	1
*SCN5A*	*SCN5A*-related disorders	*SCN5A* c.4501C>G (p.Leu1501Val), NM_198056, HET, LP	Clinical	PD	Cardiac	YES	1
*SERPINA1*	*SERPINA1*-related disorders	*SERPINA1* Pi*SZ, NM_001127701, COMP HET, ER	Clinical	PD	Organ health	NO	1
*SERPINA1* ^*a*^	*SERPINA1*-related disorders	*SERPINA1* Pi*MZ, NM_001127701, HET, ER	Clinical	PD	Organ health	NO	6
*SPINK1* ^*e*^	Susceptibility to pancreatitis	*SPINK1* c.56–37T>C, NM_003122, HET, ER	Clinical	PD	Organ health	NO	2
*TTN*	Titinopathies	*TTN* c.85640_85652del (p.Pro28547Glnfs*12), NM_001267550, HET, LP	Clinical	PD	Cardio	YES	1
*F2*	Thrombosis	*F2* c.*97G>A, NM_000506, HET, ER	Risk	PD	Clotting	NO	14
*F5*	Thrombosis	*F5* c.1601G>A (p.Arg534Gln), NM_000130, HET, ER	Risk	PD	Clotting	NO	13
All	​	​	​	​	​	​	90

VUS, variant of uncertain significance; HET, heterozygous; HOM, homozygous; ER, established risk; P, pathogenic; LP, likely pathogenic; COMP HET, compound heterozygous; HEM, hemizygous; PD, previously described; NLoF, Novel loss of function. The letter in super-index a, b, c, d, e, f and g associates patients that presented 2 variants in the clinical section.

Clinically relevant variants detected can be classified in two different categories, (1) variants related to monogenic disorders classified as P or LP, and (2) variants classified as variants of established risk. Variants included in category (1) are rare and relatively highly penetrant with an allele frequency lower than 1% and supported by clinical evidence and/or co-segregation studies. Variants in category (2) are low impact variants with an allele frequency higher than 1% and associated with common diseases. The 90 clinically relevant variants were identified in 79/400 participants (19.8%), including 11 individuals who carried two variants related to different conditions (Table 1). For patients with a clinically relevant variant, the available PFH was reviewed to establish if the patient met criteria to perform a genetic test related to the hereditary condition, or if the finding would have gone unnoticed.

**FIGURE 1:**
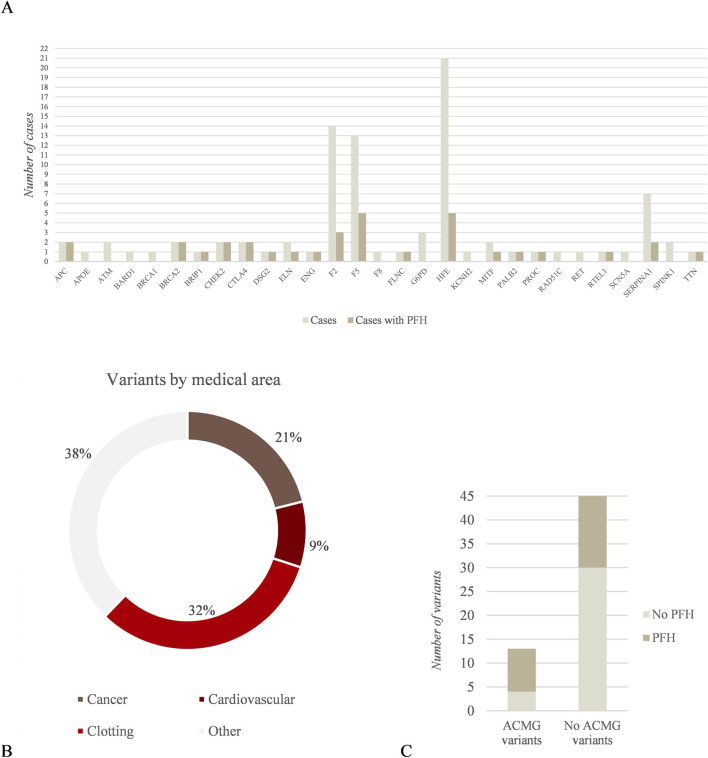
Analysis of clinically relevant variants in the analysis cohort. **(A)** Number of cases presenting clinically relevant variants segmented by gene and patients that presented PFH related to the specific phenotype; **(B)** Percentage of variants included in each medical area; **(C)** Number of cases that presented clinically relevant variants included in the ACMG SF list and the correlation with PFH (excluding F2 and F5 in the “No ACMG” category).

From the 79 individuals presenting clinically relevant variants, 28/79 (35.4%) presented variants classified in group (1) and 15/28 of them (53.6%) had PFH related to the conditions associated with the variants detected. On the other hand, 57/79 (72.2%) presented variants classified in group (2). After excluding 3 patients whose PFH was unavailable, 18/54 (33.3%) presented PFH. These data are reflected in Figure 1A. Based on medical area or pathology, among the 90 variants detected, the majority were related to clotting disorders (29/90), hereditary cancer (19/90) and cardiovascular disease (8/90) (Figure 1B). The rest of the variants were included in several medical areas (34/90). Interestingly, we identified 4 variants in X-linked genes in our cohort. These included 3 variants in the *G6PD* gene, although only 1 was hemizygous, and 1 hemizygous variant in the *F8* gene.

The variants detected in any of the 81 genes included in the ACMG SF list v3.2 (Miller et al., 2023) are indicated in Table 1. From all the participants, 13/400 (3.3%) presented variants specifically mentioned in the ACMG SF list v3.2% and 69.2% (n = 9/13) of them showed potential association with PFH (Figure 1C). Interestingly, 2 patients presented variants in genes included in ACMG SF list v3.2 together with other variants not included in the list.

### 3.2 Prevalence of clinical variants in cancer predisposition genes

The identification of pathogenic genomic variants in a healthy population offers the opportunity to identify individuals at risk for preventable cancers and other diseases who otherwise would not be under medical surveillance. Cancer screening guidelines recommend genetic testing when there is high suspicion of a hereditary cancer syndrome based on PFH. The factors considered by most expert guidelines to be more likely related to hereditary cancer risk are 1) any relative diagnosed with cancer before the age of 50 (Murff et al., 2006), 2) several relatives with cancer on the same side of the family, 3) individuals with any blood relative with a known P/LP variant in a cancer susceptibility gene (Daly et al., 2021).

In the study cohort, out of the 400 participants, 19 presented variants related to cancer predisposition. Interestingly, 12 of these 19 patients (63.2%) did not meet criteria for genetic testing, according to their PFH (Supplementary Table S1), and would have missed the opportunity for early management and prevention.

### 3.3 Genetic variants with implication for family planning

**FIGURE 2:**
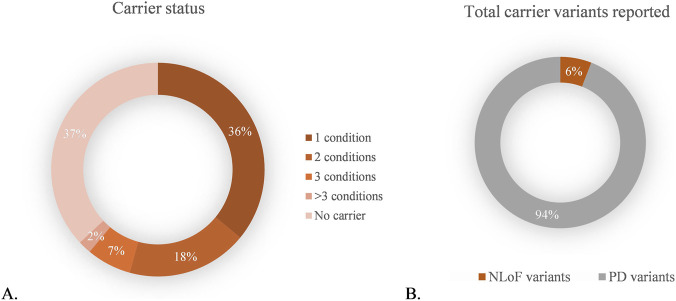
Carrier status variants in the patient cohort. **(A)** Percentage of patients carrying 1, 2, 3 or more variants related to autosomal recessive or X-linked conditions; **(B)** Percentage of new loss of function and previously described variants detected. NLoF: Novel loss of function; PD: previously described.

Variants in autosomal recessive and X-linked inheritance genes were analyzed according to ACMG guidelines. A total of 407 variants in 101 genes were detected in 252 participants (63%) who were found to be carriers of 1–5 recessive genetic conditions, with implications for reproductive screening (Figure 2A). From the 407 variants, 24 (5.9%) were NLoF (Figure 2B; the list of variants is available in Supplementary Table S2). Interestingly, 8 of the 24 NLoF variants detected and reported as LP, were included in ClinVar in the last 2 years.

Carrier frequency in the study cohort was compared with the data available in published articles for some conditions that are included in regular carrier screening testing, following the American College of Obstetricians and Gynaecologists (ACOG) recommendations (Taber et al., 2022) (Supplementary Table S3). Although the cohort is small, the carrier frequency for most of the conditions was consistent with published evidence.

### 3.4 Risk variants related to multifactorial diseases

A risk allele refers to a variant with low penetrance for which the associated risk does not present a Mendelian inheritance pattern but is present more frequently in affected individuals than in healthy controls (van Rossum et al., 2005). To evaluate which risk alleles should be included in the clinical report, the company established a procedure based on objective data elements, assigning them a weight to assess the inclusion or exclusion of new risk variants and conditions (Senol-Cosar et al., 2019).

**FIGURE 3:**
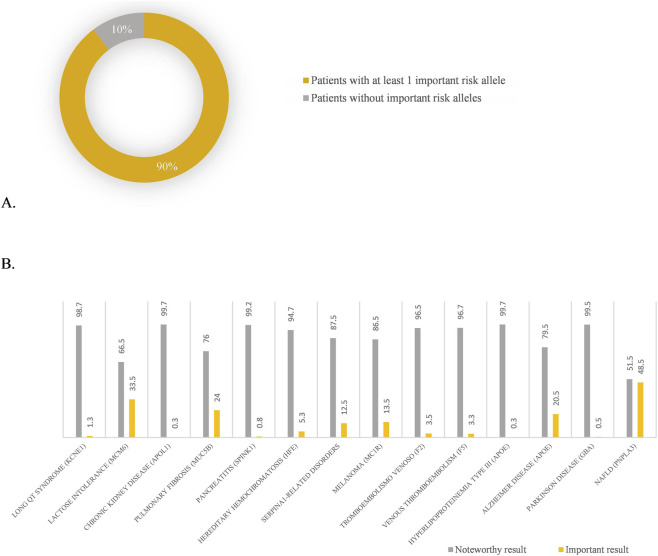
Risk alleles detected on the analysed population. **(A)** Percentage of the patients that presented at least one important risk allele. **(B)** Percentage of participants that presented each one of the risk alleles. NAFLD: Non-Alcoholic Fatty Liver Disease. Note: The table reflects only those pathologies in which risk variants have been identified; variants classified as clinically relevant are not displayed on the graph. There are several risk alleles excluded from the table because no variant was detected in our cohort.

In our cohort, risk alleles related to 15 conditions were analyzed and 89.8% of individuals were carriers of at least one risk allele (Figure 3A). Although the risk variants on their own are not expected to cause disease, they imply a well described risk that interacts with environmental factors to trigger the condition. Knowing this information in advance can aid the implementation of preventive strategies or the adaptation of modifiable factors to reduce the overall patient risk.

Risk alleles related to non-alcoholic fatty liver disease, lactose intolerance, pulmonary fibrosis and Alzheimer disease were the most common risk variants observed in the 400 patients, with detection rates of 48.8%, 33.5%, 24% and 20.5% of the individuals respectively (Figure 3B). Variants related to melanoma and hemochromatosis were also found in 13.5% and 5.3% of the participants respectively.

### 3.5 Prevalence of *F2* and *F5* established clinical risk

Factor II (*F2*) c.*97G>A and Factor V Leiden (*F5*) c.1601G>A are examples of risk alleles with clinical relevance. Current strategies for preventing venous thromboembolism (VTE) fail to address primary prevention in the general population (Folsom and Cushman, 2020). By knowing the genetic risk in advance, primordial prevention of unprovoked VTE would involve education, environmental changes, the reduction of modifiable VTE risk factors (Folsom et al., 2023; Lutsey and Zakai, 2023) or even a short course of prophylactic anticoagulation when transient risk factors are present (Pastori et al., 1999).

The frequency of the *F2* and *F5* variants in the study cohort was 3.5% and 3.3% respectively. Allele frequency in European population for *F2* c.*97G>A is approximately 1.7%–3% (Rosendaal et al., 1998; Kujovich, 2006) and 3%–8% for *FV* Leiden (Kujovich, 2011), which is consistent with the allele frequency observed in the study cohort. Overall, 27 of the 400 individuals (6.8%) carried either the *F2* c.*97G>A or the *F5* Leiden c.1601G>A risk alleles. Among the 27 individuals, 24 had available PFH and 33.3% of those presented with PFH related to thrombophilia (Supplementary Figure S1).

### 3.6 Prevalence of clinical variants associated with hereditary haemochromatosis

The main genotype associated with the common form of adult haemochromatosis (HH) is the homozygosity for the p.Cys282Tyr (C282Y) mutation in the *HFE* gene. This mutation is frequent in individuals of European descent, where one in five to ten are carriers (Cheng et al., 2009; Coppin et al., 2003). The ACMG SF list v3.2 includes the *HFE* mutation C282Y in the homozygous state (Miller et al., 2023) as a secondary finding to be reported in opportunistic screening. However, since there are patients with other genotypes described in the literature related to HH (Hou et al., 2020; Sonagra and Zubair, 2023; Mottelson et al., 2024), the pipeline was designed to include *HFE* C282Y and H63D variants, as they are also related with Type 1 HH, the most common cause of iron overload (Kowdley et al., 2019).

In our cohort, no homozygous *HFE* C282Y genotype was observed. However, 21 individuals were detected with other non-ACMG SF list v3.2 genotypes included in our study (Supplementary Figure S2A). After examination of participant’s clinical history, a potential genotype-phenotype association was found in 5 cases (25%) (Supplementary Figure S2B).

### 3.7 Pharmacogenomic variants with implication in drug prescription

**FIGURE 4:**
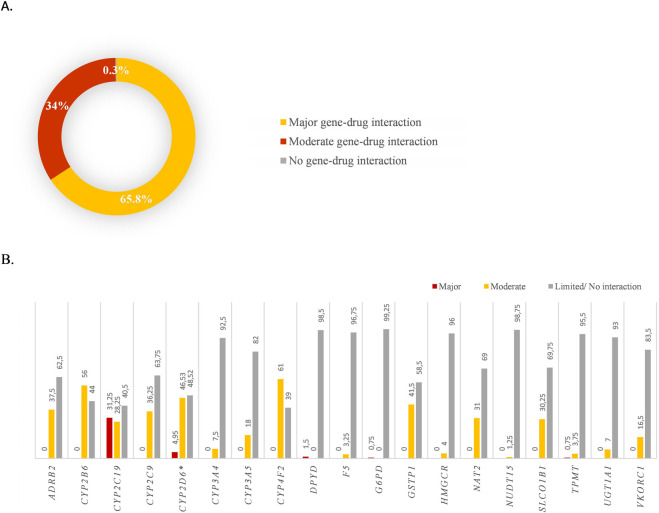
Pharmacogenomic variants present in the analysed population. **(A)** Percentage of patients with Major and Moderate gene-drug interaction variants. Note: the patients represented with major gene-drug interactions also presented moderate gene-drug interactions; Patients indicated as having moderate gene-drug interactions only presented this type of results; **(B)** Percentage of patients that presented variants specified by gene.

The pharmacogenetic evaluation included 27 genes that affect drug metabolism, transport or targets. Of the 400 participants, 34% presented a variant with a severe gene-drug interaction, 65.8% presented at least one variant with a moderate drug-gene interaction, thus 99.8% of the cohort harboured at least one allele related to drug prescription, which is consistent with previous studies in the Spanish population (Nunez-Torres et al., 2023) (Figure 4A).

The most common gene-drug interaction was related to haematology and cardiovascular disease. Other gene-drug interactions detected in the cohort were associated with oncology, pain medicine, transplant medicine, and immunological disorders (Supplementary Table S4). The *CYP2C19*-Clopidogrel severe interaction was present in 31.3% of individuals (Figure 4B). The *CYP2D6* gene was only analysed in 101 individuals (since it was included later in the analysis), 5% of them showed severe and moderate gene-drug interactions and 46.5% showed only moderate gene-interactions related to this gene (Figure 4B).

## 4 Discussion

We present the results of the first hospital checkup unit offering preventive genomic screening for actionable adult-onset diseases as a standard of care, integrating it with other biochemical and image testing. The EGT included the analysis of over 560 genes and 19.8% of participants in our cohort presented clinically relevant variants. These variants, primarily associated with conditions such as hemochromatosis, cancer predisposition, cardiovascular diseases, endocrine and metabolic conditions, and clotting disorders, triggered the adaptation of patient medical management and/or additional testing. This approach provided a higher rate of at-risk patients compared to other genomic screening strategies published to date. The most likely cause is the expanded selection of actionable disease-related genomic aspects included in our screen, relative to other publications.

The analysis focused on aspects of interest related to preventive genomics, such as adult-onset monogenic diseases, risk alleles, carrier status and pharmacogenomic profiling. These four aspects of genomics have been discussed and proved clinically useful for the general population in different studies, providing actionable information to practically all patients attending the unit.

Of the 400 patients, 13 individuals (3.3%) presented with clinical variants included in the ACMG SF list v3.2. This data is highly consistent with recently published data based on a large cohort of patients, which suggests that around 3.4% of the population may present a variant included in this list (Savatt et al., 2025). This percentage may be even higher according to publications based on previous versions of the list (Carmona et al., 2024; Miller et al., 2021; Johnston et al., 2022).

Taking into account that 38.9% of the patients detected with variants related to hereditary cancer in our cohort did not present any PFH, it must be considered that current clinical practice is missing the opportunity of prevention or early detection in a significant number of individuals. Similarly, for patients presenting variants related to cardiovascular hereditary disease 42.9% did not present PFH.

Carrier screening testing is recommended to be offered to all couples (Committee on Genetics, 2017), regardless of their ethnicity, as 3% of them are at risk of having a child with a recessive condition (Ben-Shachar et al., 2019). Prior awareness of parental carrier status can facilitate informed decisions regarding family planning. Considering the current technical and bioinformatic limitations inherent to whole genome sequencing (WGS) analysis, certain diseases with complex molecular background cannot be analyzed accurately (Guha et al., 2024). Specific genes presenting pseudogenes are challenging to analyze through next-generation sequencing due to the high homology between the pseudogene and the functional parental gene; consequently, conditions such as congenital adrenal hyperplasia (*CYP21A2* gene) or spinal muscular atrophy (*SMN1* gene) require additional methodology to provide reliable results. Another variant type that poses a challenge for this technology when using short-reads sequencing, are repeat expansions related to conditions such as Fragile-X syndrome (*FMR1* gene). In this case, the restricted read length and the repetitive nature of the variants leads to ambiguous alignment and mapping errors. Currently, novel bioinformatic analysis tools are being validated to overcome this limitation such as Expansion Hunter (Dolzhenko et al., 2017). It is reasonable to expect that in the near future, the analysis of technically challenging conditions from WGS data will be possible, enabling the reporting of a comprehensive WGS based clinical carrier screening.

Pharmacogenomics profile has proven not only to be clinically useful to adapt medical prescription, but there is growing evidence suggesting it can also be cost-effective for relevant selected drug-gene interactions (Morris et al., 2022). Around 34% of the participants presented a variant responsible for severe drug-gene interaction that justifies the adaptation of drug prescription (Supplementary Table S4). The most common severe drug interaction in our cohort was the presence of loss of function *CYP2C19* alleles related to clopidogrel prescription associated with major adverse cardiovascular events after a percutaneous coronary intervention when clopidogrel is prescribed (Mega et al., 2010). In our population, 125 patients (31.25%) presented a variant related to a severe drug-gene interaction impacting the prescription of this drug. This percentage is consistent with the expected 30% of people affected based on previous publications in the Spanish population (Nunez-Torres et al., 2023).

Due to the relevance of the side effects, it is worth mentioning that 6 patients (1.5%) from our cohort presented variants in the *DPYD* gene, related to oncology treatment. The study of this gene is recommended by the European Medicines Agency because up to 9% of the Caucasian population presents a partial deficiency (EMA, 2020); the initial dose must be adapted in those patients. In case of complete deficiency capecitabine, fluorouracil or tegafur must not be prescribed because of life-threatening side effects (Amstutz et al., 2018).

Regarding multifactorial diseases, in our cohort we found that the prevalence of *F2* and *F5* variants related to thrombophilia susceptibility are concordant with previous publications. These risk alleles are easily actionable and allow the implementation of prevention strategies for many patients. HH is one of the most common disorders among individuals of northern European descent (Kowdley et al., 2019). We found 3 participants in our cohort presenting *HFE* compound heterozygous C282Y/H63D mutations, which are related to HH Type 1B. Although this genotype represents a phenotype with lower clinical expression than Type 1A (C282Y in the homozygous state), it can still cause iron overload and therefore these patients require treatment and follow-up.

The patient ancestry information was also reported; this data is useful to understand if the patient belongs to an ancestry group that might be related to specific founder P or LP variants, common in certain diseases. Ancestry determination can also be relevant for future applications such as a more accurate assessment of polygenic risk score adjusted by ethnicity.

**FIGURE 5:**
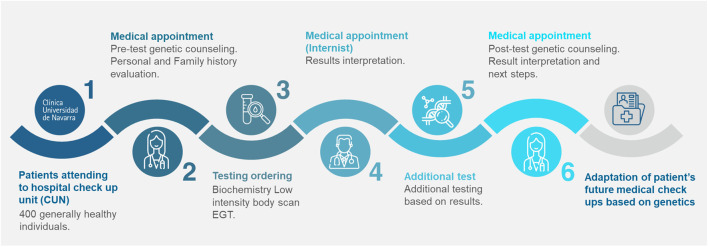
Hospital genomic checkup workflow. CUN: Clinica Universidad de Navarra; EGT: Elective genomic testing.

In the genomic era the integration of EGT in a hospital checkup unit seems a natural step forward to provide a personalized approach for preventive medicine. Genomic information has been the missing piece of the preventive screening puzzle, the aim of this publication is to provide insight into the potential benefits of including EGT in the clinical practice of a checkup unit, allowing the opportunity of tailoring the screening strategy of patients attending the unit (Figure 5).

This study has two primary limitations: the restricted cohort size and the absence of patient follow-up due to sporadic attendance at the check-up unit. Although individuals from across Spain and other countries visit the consultation annually for check-ups, some patients do not return for several years, making long-term follow-up largely unavailable. Most of the conditions included in the analysis are adult-onset diseases, therefore signs and symptoms related to genetic variants may be absent at the time of testing. The lack of follow-up makes it difficult to understand the long-term impact of this enhanced screening in patients’ health. Furthermore, since the penetrance of genetic variants in a healthy setting may vary from that in a diagnostic environment, further investigation would be useful to have a better and more precise understanding of the penetrance in this population.

Although long term patient follow up is generally not available in our cohort, the inclusion of EGT in the checkup unit triggered the realization of additional follow up testing based on genetic results, and the adaptation of the drug prescription in many patients. Furthermore, signs and symptoms may not be present at the time of testing, but the results provide valuable insights to segment at risk individuals for a surveillance strategy throughout their lives. There is growing evidence on proactive genomic screening in different settings, our results provide initial evidence to assess the potential of a comprehensive approach integrating EGT into a checkup unit.

The cost of whole genome sequencing has fallen significantly in recent years, triggering a surge in neonatal genomic screening initiatives in various countries (Lunke et al., 2024; Kingsmore and BeginNGS Consortium, 2022; Ferlini et al., 2023). In this regard, it seems reasonable to think that screening for adult-onset diseases is equally important, and it is worth further attention. There is published evidence about the cost-effectiveness of genetic screening for hereditary cancer (Bourke et al., 2025) and the population screening for a group of Tier1 CDC conditions (Guzauskas et al., 2023), suggesting that a broader screening could also meet the conditions to be incorporated into a health system, balancing savings and costs, improving patient’s quality and duration of life.

The genomic findings collected in this study highlight the clinical relevance of genetic screening as a preventive medicine tool, both for the prevention or early detection of hereditary diseases and for the adaptation of drug prescription in the general population. To implement population genomic screening effectively and equitably, there are strategies that can be put in place, such as establishment of a multi-institutional genomic screening consortium that ensures the representation of diverse ethnicities and populations, creation of a framework to define and update which genes and variants should be analyzed, and the design and evaluation of consensus-based multilevel interventions based on genetic findings (Buchanan et al., 2024).

## Data Availability

The original contributions presented in the study are included in the article/Supplementary Material, further inquiries can be directed to the corresponding author.
