# Supplementary material for: Elective genomic screening: results of the implementation of a whole genome sequencing program at a medical check-up unit in Spain
Source: Front Genet. 2026 Jan 23;16:1722462. doi: 10.3389/fgene.2025.1722462 (PMC12877404; doi:10.3389/fgene.2025.1722462)
Supplement: Supplementary file 1 [file DataSheet1.pdf]

Supplementary Material

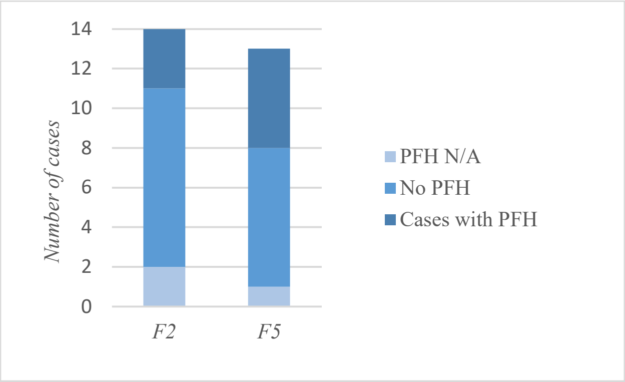

Supplementary Figure S1. Patients of the cohort that presented risk alleles in *F2* or *F5* genes and the association with personal or family history (PFH). N/A: Not available.

Patient num	Age	F/M	Gene	Condition	Variant	PH/FH	Details	Age onset <50	Meet criteria for genetic testing
1	37	F	*ATM*	Cancer susceptibility related to *ATM* variants	*ATM* c.329_330delGA (p.Arg110Lysfs*4), NM_000051, HET, LP	No	N/A	N/A	No
2	59	M	*ATM*	Cancer susceptibility related to *ATM* variants	*ATM* c.5188C>T (p.Arg1730*), NM_000051, HET, LP	No	N/A	N/A	No
3	43	F	*BARD1*	Cancer susceptibility related to *BARD1* variants	*BARD1* c.1728_1731dup (p.Ser578Alafs*11), NM_000465, HET, LP	No	N/A	N/A	No
4	65	M	*BRCA1*	Hereditary Breast and Ovarian Cancer Syndrome	*BRCA1* c.5152+2T>G (p.?), NM_007294, HET, LP	No	N/A	N/A	No
5	67	M	*BRCA2*	Hereditary Breast and Ovarian Cancer Syndrome	*BRCA2* c.2808_2811del (p.Ala938Profs*21), NM_000059, HET, P	Yes	FH: First degree relative breast cancer at the age of 20	Yes	Yes
6	49	M	*BRCA2*	Hereditary Breast and Ovarian Cancer Syndrome	*BRCA2* c.2808_2811del (p.Ala938Profs*21), NM_000059, HET, P	Yes	FH: Mother carcinoma of unknown origin	N/A	No
7	53	M	*BRIP1*	Cancer susceptibility related to *BRIP1*	*BRIP1* c.1702_1703delAA (p.Asn568Trpfs*9), NM_032043, HET, P	Yes	FH: Father bladder cancer	N/A	No
8	59	M	*CHEK2*	Cancer susceptibility related to *CHEK2*	*CHEK2* c.1100delC (p.Thr367Metfs*15), NM_007194, HET, P	Yes	FH: Father lung cancer; Mother breast cancer	N/A	Yes
9	45	M	*CHEK2*	Cancer susceptibility related to *CHEK2*	*CHEK2* c.319+2T>A, NM_007194, HET, LP	Yes	PH: Colon and renal cancer<br>FH: Mother breast cancer; Sister endometrial cancer	N/A	Yes
10	32	M	*CTLA4*	Autoimmune Lymphoproliferative Syndrome	*CTLA4* c.160G>A (p.Ala54Thr), NM_005214, HET, VUS with solid evidence	Yes	FH: Brother lymphoproliferative syndrome; Mother CTLA4 gene carrier	No	Yes
11	58	F	*CTLA4*	Autoimmune Lymphoproliferative Syndrome	*CTLA4* c.160G>A (p.Ala54Thr), NM_005214, HET, VUS with solid evidence	Yes	FH: Son lymphoma	Yes	Yes
12	48	M	*PALB2*	*PALB2*-predisposition to cancer	*PALB2* c.1240C>T (p.Arg414*), NM_024675, HET, P	Yes	FH: Mother colon cancer	No	No
13	43	F	*RAD51C*	Ovarian cancer	*RAD51C* c.404G>A (p.Cys135Tyr), NM_058216, HET, LP	No	N/A	N/A	No
14	68	M	*RET*	Multiple endocrine neoplasia	*RET* c.2410G>A (p.Val804Met), NM_020975, HET, P	No	N/A	N/A	No
15	50	F	*RTEL1*	*RTEL1*-related disorders	*RTEL1* c.2956C>T (p.Arg986*), NM_016434, HET, P	Yes	FH: Father bladder and prostate cancer	N/A	No
16	66	M	*APC*	Colorectal cancer	*APC* c.3920T>A (p.Ile1307Lys), NM_000038, HET, ER	Yes	PH: Mediastinal carcinoid<br>FH: Mother breast cancer; Sister breast and lung cancer	N/A	Yes
17	51	M	*APC*	Colorectal cancer	*APC* c.3920T>A (p.Ile1307Lys), NM_000038, HET, ER	Yes	PH: Adenomatous polyp with low grade dysplasia<br>FH: Mother gastric cancer	N/A	Yes
18	41	M	*MITF*	Cutaneous melanoma	*MITF* c.1255G>A (p.Glu419Lys), NM_198159, HET, ER	Yes	FH: Mother colon cancer	N/A	No
19	51	M	*MITF*	Cutaneous melanoma	*MITF* c.1255G>A (p.Glu419Lys), NM_198159, HET, ER	No	N/A	N/A	No

Supplementary Table S1. Analysis of family and personal history in patients with cancer related variants. F: Female; M: Male; HET: Heterozygous; HOM: Homozygous; ER: Established Risk; P: Pathogenic; LP: Likely Pathogenic; VUS: Variants of unknown significance; PH: Personal history; FH: Family history; N/A: Data not available.

A.

HFE Variants	Number of variants
C282Y Het	18
Comp Het (H63D; C282Y)	3

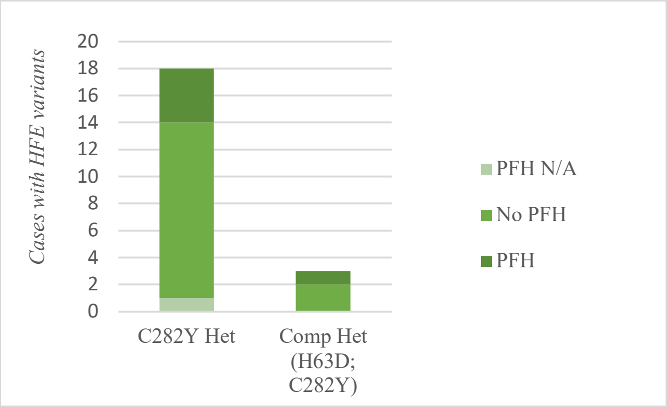

B.

Supplementary Figure S2. Variants related to hemochromatosis phenotype. A) Variants identified on the analysis cohort, B) Analysis of personal and family history in patients with variants related with hemochromatosis. Het: Heterozygous; Comp Het: Compound Heterozygous; PFH: Personal or Family History; N/A: Not available.

Variant	Variant type
ABCC8 c.62T>A (p.Val21Asp), NM_000352, Het, LP	PD
ABCG8 c.1083G>A (p.Trp361*), NM_022437, Het, P	PD
ABCG8 c.1608G>A (p.Trp536*), NM_022437, Het, LP	PD
ACADM c.1189dupT (p.Tyr397Leufs*5), NM_000016, Het, LP	PD
ACADM c.443G>A (p.Arg148Lys), NM_000016, P	PD
ACADM c.600-18G>A, NM_000016, Het, LP	PD
ACADM c.600-18G>A, NM_000016, Het, LP	PD
ACADM c.820A>G (p.Met274Val), NM_000016, Het, LP	PD
ACADM c.997A>G (p.Lys333Glu), NM_001127328, Het, P	PD
ACADM c.997A>G (p.Lys333Glu), NM_001127328, Het, P	PD
ADACM c.106G>T (p.Gly36*), NM_000016, Het, LP	NLoF
ACADSB c.1159G>A (p.Glu387Lys), NM_001609, Het, LP	PD
ACADSB c.443C>T (p.Thr148Ile), NM_001609, Het, P	PD
ACADSB c.621G>A (p.Trp207*), NM_001609, Het, LP	PD
ACADVL c.1153C>T (p.Arg385Trp), NM_000018, Het, LP	PD
ACSF3 c.1075G>A (p.Glu359Lys), NM_174917, Het, P	PD
ACSF3 c.1075G>A (p.Glu359Lys), NM_174917, Het, P	PD
AGL c.1571G>A (p.Arg524His), NM_000642, Het, LP	PD
AGL c.3216_3217delGA (p.Glu1072Aspfs*36), NM_000642, Het, LP	PD
AGL c.3652C>T (p.Arg1218*), NM_000642, Het, P	PD
ALDOB c.448G>C (p.Ala150Pro), NM_000035, Het, P	PD
ALDOB c.448G>C (p.Ala150Pro), NM_000035, Het, P	PD
ALDOB c.448G>C (p.Ala150Pro), NM_000035, Het, P	PD
ALDOB c.448G>C (p.Ala150Pro), NM_000035, Het, P	PD
ALDOB c.524C>A (p.Ala175Asp), NM_000035, Het, P	PD
ALDOB c.911G>A (p.Arg304Gln), NM_000035, Het, LP	PD
ALDOB c.911G>A (p.Arg304Gln), NM_000035, Het, LP	PD
ALDOB c.911G>A (p.Arg304Gln), NM_000035, Het, LP	PD
ALPL c.407G>A (p.Arg136His), NM_000478.4, Het, P	PD
ALPL c.571G>A (p.Glu191Lys), NM_000478, Het, P	PD
ALPL c.809G>A (p.Trp270*), NM_000478, Het, P	PD
ARSA c.769G>C (p.Asp257His), NM_000487, Het, P	PD
ATM c.5188C>T (p.Arg1730*), NM_000051, Het, LP	PD
ATP7B c.1934T>G (p.Met645Arg), NM_000053, Het, P	PD
ATP7B c.1934T>G (p.Met645Arg), NM_000053, Het, P	PD
ATP7B c.1934T>G (p.Met645Arg), NM_000053, Het, LP	PD
ATP7B c.1934T>G (p.Met645Arg), NM_000053, Het, LP	PD
ATP7B c.1934T>G (p.Met645Arg), NM_000053, Het, LP	PD
ATP7B c.1934T>G (p.Met645Arg), NM_000053, Het, LP	PD
ATP7B c.1934T>G (p.Met645Arg), NM_000053, Het, LP	PD
ATP7B c.1934T>G (p.Met645Arg), NM_000053, Het, LP	PD
ATP7B c.2304dupC (p.Met769Hisfs*26), NM_000053, Het, P	PD
ATP7B c.2532delA (p.Val845Serfs*28), NM_000053, Het, P	PD
ATP7B c.2605G>A (p.Gly869Arg), NM_000053, Het, LP	PD
ATP7B c.2605G>A (p.Gly869Arg), NM_000053, Het, LP	PD
ATP7B c.2605G>A (p.Gly869Arg), NM_000053, Het, LP	PD
ATP7B c.3182G>A (p.Gly1061Glu), NM_000053, Het, P	PD

Variant	Variant type
GJB2 c.101T>C (p.Met34Thr), NM_004004, Het, P	PD
GJB2 c.101T>C (p.Met34Thr), NM_004004, Het, P	PD
GJB2 c.167delT (p.Leu56Argfs*26), NM_004004, Het, LP	PD
GJB2 c.-22-2A>C (p.?), NM_004004, Het, LP	PD
GJB2 c.269dupT (p.Val91Serfs*11), NM_004004, Het, P	PD
GJB2 c.30delG (p.Gly12Valfs*2), NM_004004, Het, P	PD
GJB2 c.35delG (p.Gly12Valfs*2), NM_004004, Het, P	PD
GJB2 c.35delG (p.Gly12Valfs*2), NM_004004, Het, P	PD
GJB2 c.35delG (p.Gly12Valfs*2), NM_004004, Het, P	PD
GJB2 c.35delG (p.Gly12Valfs*2), NM_004004, Het, P	PD
GJB2 c.35delG (p.Gly12Valfs*2), NM_004004, Het, P	PD
GJB2 c.35delG (p.Gly12Valfs*2), NM_004004, Het, P	PD
GJB2 c.439G>A (p.Glu147Lys), NM_004004, Het, P	PD
GJB2 c.617A>G (p.Asn206Ser), NM_004004, Het, P	PD
GJB2 c.617A>G (p.Asn206Ser), NM_004004, Het, P	PD
GJB2 c.617A>G (p.Asn206Ser), NM_004004, Het, P	PD
GJB2 c.617A>G (p.Asn206Ser), NM_004004, Het, P	PD
GJB2 c.617A>G (p.Asn206Ser), NM_004004, Het, P	PD
GLB1 c.1745G>A (p.Trp582*), NM_000404, Het, LP	NLoF
HADHA c.1528G>C (p.Glu510Gln), NM_000182, Het, P	PD
HADHA c.1528G>C (p.Glu510Gln), NM_000182, Het, P	PD
HADHA c.1981_1999del (p.Leu661Serfs*12), NM_000182, Het, LP	PD
HADHA c.1981_1999del (p.Leu661Serfs*12), NM_000182, Het, LP	PD
HBB c.92+6T>C, NM_000518, Het, P	PD
HBB c.93-21G>A (p.?), NM_000518, Het, P	PD
HEXA c.745C>T (p.Arg249Trp), NM_000520, Het, No known risk	PD
HEXB c.1250C>T (p.Pro417Leu), NM_000521, Het, P	PD
ITK c.851+1G>T, NM_005546, Het, LP	NLoF
IVD c.102_103del (p.Val35Glyfs*20), NM_002225, Het, LP	PD
IVD c.941C>T (p.Ala314Val), NM_002225, Het, P	PD
KCNQ1 c.898G>A (p.Ala300Thr), NM_000218, Het, LP	PD
LAMA2 c.1854_1861dupACGTGTTC (p.Leu621Hisfs*7), NM_000426, Het, P	PD
LAMA2 c.2049_2050delAG (p.Arg683Serfs*21), NM_000426, Het, P	PD
LAMA2 c.2187delG (p.Tyr730Ilefs*45), NM_000426, Het, LP	NLoF*
LAMA2 c.4739dupG (p.Leu1581Profs*5), NM_000426, Het, P	PD
LMNA c.892C>T (p.Arg298Cys), NM_170707, Het, P	PD
LPL c.755T>C (p.Ile252Thr), NM_000237, Het, P	PD
LRPPRC c.1842+1G>A, NM_133259, Het, LP	NLoF
LTBP2 c.5320+1G>T, NM_000428, Het, LP	NLoF
MEFV c.2082G>A (p.Met694Ile), NM_000243, Het, P	PD
MEFV c.2082G>A (p.Met694Ile), NM_000243, Het, P	PD
MEFV c.2230G>T (p.Ala744Ser), NM_000243, Het, P	PD
MEFV c.2230G>T (p.Ala744Ser), NM_000243, Het, P	PD
MEFV c.2230G>T (p.Ala744Ser), NM_000243, Het, P	PD
MEFV c.2230G>T (p.Ala744Ser), NM_000243, Het, P	PD
MEFV c.2230G>T (p.Ala744Ser), NM_000243, Het, P	PD
MEFV c.2230G>T (p.Ala744Ser), NM_000243, Het, P	PD

BBS1 c.1169T>G (p.Met390Arg), NM_024649, Het, P	PD
BCHE c.293A>G (p.Asp98Gly), NM_000055, Het, P	PD
BCHE c.293A>G (p.Asp98Gly), NM_000055, Het, P	PD
BCHE c.293A>G (p.Asp98Gly), NM_000055, Het, P	PD
BCHE c.293A>G (p.Asp98Gly), NM_000055, Het, P	PD
BCHE c.293A>G (p.Asp98Gly), NM_000055, Het, P	PD
BCHE c.293A>G (p.Asp98Gly), NM_000055, Het, P	PD
BCHE c.293A>G (p.Asp98Gly), NM_000055, Het, P	PD
BCHE c.293A>G (p.Asp98Gly), NM_000055, Het, P	PD
BCHE c.293A>G (p.Asp98Gly), NM_000055, Het, P	PD
BCHE c.293A>G (p.Asp98Gly), NM_000055, Het, P	PD
BCHE c.293A>G (p.Asp98Gly), NM_000055, Het, P	PD
BCHE c.293A>G (p.Asp98Gly), NM_000055, Het, P	PD
BCHE c.293A>G (p.Asp98Gly), NM_000055, Het, P	PD
BCHE c.293A>G (p.Asp98Gly), NM_000055, Het, P	PD
BCHE c.293A>G (p.Asp98Gly), NM_000055, Het, P	PD
BCHE c.293A>G (p.Asp98Gly), NM_000055, Het, P	PD
BCHE c.293A>G (p.Asp98Gly), NM_000055, Hom, P	PD
BCHE c.435delTinsAG (p.Phe146Valfs*12), NM_000055, Het, P	PD
BCHE c.812C>T (p.Thr271Met), NM_000055, Het, LP	PD
BCHE c.895G>T(p.Glu299*), NM_000055, Het, LP	PD
BCKDHB c.348delA (p.Asp117Ilefs*113), NM_000056, Het, P	PD
BRCA1 c.5152+2T>G (p.?), NM_007294, Het, LP	PD
BRCA2 c.2808_2811del (p.Ala938Profs*21), NM_000059, Het, P	PD
BRCA2 c.2808_2811delACAA (p.Ala938Profs*21), NM_000059, Het, P	PD
BRIP1 c.1702_1703delAA (p.Asn568Trpfs*9), NM_032043, Het, P	PD
BTD c.1429C>T (p.Pro477Ser), NM_001370658, Het, P	PD
BTD c.535G>A (p.Val179Met), NM_001370658, Het, P	PD
CACNA1S c.541+2T>A, NM_000069, Het, LP	NLoF
CBS c.1330G>A (p.Asp444Asn), NM_000071, Het, P	PD
CERKL c.769C>T (p.Arg257*), NM_201548, Het, P	PD
CERKL c.769C>T (p.Arg257*), NM_201548, Het, P	PD
CFTR c.[220C>T(|)601G>A(|)3808G>A] (p.[Arg74Trp(|)Val201Met(|)Asp1270Asn]), NM_000492, Het, LP	PD
CFTR c.[220C>T(|)601G>A(|)3808G>A] (p.[Arg74Trp(|)Val201Met(|)Asp1270Asn]), NM_000492, Het, LP	PD
CFTR c.[220C>T(|)601G>A(|)3808G>A] (p.[Arg74Trp(|)Val201Met(|)Asp1270Asn]), NM_000492, Het, LP	PD
CFTR c.1210-34TG[11]T[5], NM_000492, Het, ER	PD
CFTR c.1210-34TG[11]T[5], NM_000492, Het, ER	PD
CFTR c.1210-34TG[11]T[5], NM_000492, Het, ER	PD
CFTR c.1210-34TG[11]T[5], NM_000492, Het, ER	PD
CFTR c.1210-34TG[11]T[5], NM_000492, Het, ER	PD
CFTR c.1210-34TG[11]T[5], NM_000492, Het, ER	PD
CFTR c.1327G>T (p.Asp443Tyr), NM_000492, Het, LP	PD
CFTR c.1521_1523delCTT (p.Phe508del), NM_000492, Het, P	PD
CFTR c.1521_1523delCTT (p.Phe508del), NM_000492, Het, P	PD
CFTR c.1624G>T (p.Gly542*), NM_000492, Het, P	PD
CFTR c.1624G>T (p.Gly542*), NM_000492, Het, P	PD
CFTR c.2900T>C (p.Leu967Ser), NM_000492, Het, LP	PD
MMUT c.2026G>A (p.Ala676Thr), NM_000255, Het, LP	PD
MMUT c.210_213dup (p.Leu72Thrfs*13), NM_000255, Het, LP	NLoF*
MMUT c.671_678dup (p.Val227Asnfs*16),NM_000255, Het, P	PD
MPL c.1653+1del, NM_005373, Het, LP	PD
MSH3 c.2544-1G>A, NM_002439, Het, LP	NLoF*
MUTYH c.1187G>A (p.Gly396Asp), NM_001128425, Het, P	PD
MUTYH c.1187G>A (p.Gly396Asp), NM_001128425, Het, P	PD
MUTYH c.1187G>A (p.Gly396Asp), NM_001128425, Het, P	PD
MUTYH c.1187G>A (p.Gly396Asp), NM_001128425, Het, P	PD
MUTYH c.1187G>A (p.Gly396Asp), NM_001128425, Het, P	PD
MUTYH c.536A>G (p.Tyr179Cys), NM_001128425, Het, P	PD
MUTYH c.536A>G (p.Tyr179Cys), NM_001128425, Het, P	PD
MUTYH c.536A>G (p.Tyr179Cys), NM_001128425, Het, P	PD
MYO7A c.5618G>A (p.Arg1873Gln), NM_000260, Het, P	PD
MYO7A c.5944G>A (p.Gly1982Arg), NM_000260, Het, LP	PD
MYO7A c.6025delG (p.Ala2009Profs*32) ,NM_000260, Het, P	PD
NTHL1 c.268C>T (p.Gln90*), NM_002528, Het, P	PD
NTHL1 c.350delC (p.Pro117Glnfs*12), NM_002528, Het, LP	PD
PAH c.1066-11G>A (p.?), NM_000277, Het, P	PD
PAH c.1208C>T (p.Ala403Val), NM_000277, Het, P	PD
PAH c.1208C>T (p.Ala403Val), NM_000277, Het, P	PD
PAH c.1208C>T (p.Ala403Val), NM_000277, Het, P	PD
PAH c.194T>C (p.Ile65Thr), NM_000277, Het, P	PD
PAH c.261C>A (p.Ser87Arg), NM_000277, Het, LP	PD
PAH c.688G>A (p.Val230Ile), NM_000277, Het, P	PD
PAH c.838G>A (p.Glu280Lys), NM_000277, Het, P	PD
PAH c.842C>T (p.Pro281Leu), NM_000277, Het, LP	PD
PALB2 c.1240C>T (p.Arg414*), NM_024675, Het, LP	PD
PEX6 c.1802G>A (p.Arg601Gln), NM_000287, Het, LP	PD
PEX6 c.1802G>A (p.Arg601Gln), NM_000287, Het, LP	PD
PEX6 c.462delG (p.Leu155Trpfs*3), NM_000287, Het, LP	PD
PKHD1 c.9689delA (p.Asp3230Valfs*34), NM_138694, Het, P	PD
PKHD1 c.9689delA (p.Asp3230Valfs*34), NM_138694, Het, P	PD
PLOD1 c.486dupC (p.Asn163Glnfs*54), NM_000302, Het, LP	NLoF*
PLOD1 c.486dupC (p.Asn163Glnfs*54), NM_000302, Het, LP	NLoF*
PMM2 c.-167G>T, NM_000303, Het, LP	PD
PMM2 c.422G>A (p.Arg141His), NM_000303, Het, P	PD
PMM2 c.422G>A (p.Arg141His), NM_000303, Het, P	PD
PMM2 c.422G>A (p.Arg141His), NM_000303, Het, P	PD
PMM2 c.422G>A (p.Arg141His), NM_000303, Het, P	PD
PMM2 c.710C>G (p.Thr237Arg), NM_000303, Het, P	PD
PMM2 c.710C>T (p.Thr237Met), NM_000303, Het, P	PD
PMM2c.422G>A (p.Arg141His), NM_000303, Het, P	PD
POLG c.1760C>T(|)752C>T (p.Pro587Leu(|)Thr251Ile), NM_002693, Het, P	PD
POLG c.1760C>T(|)752C>T (p.Pro587Leu(|)Thr251Ile), NM_002693, Het, P	PD
PRF1 c.140G>T (p.Gly47Val), NM_001083116, Het, LP	PD
PRF1 c.50delT (p.Leu17Argfs*34), NM_005041, Het, P	PD

CFTR c.2900T>C (p.Leu967Ser), NM_000492, Het, LP	PD
CFTR c.2900T>C (p.Leu967Ser), NM_000492, Het, LP	PD
CFTR c.2900T>C (p.Leu967Ser), NM_000492, Het, LP	PD
CFTR c.350G>A (p.Arg117His), NM_000492, Het, P	PD
CFTR c.3717+1G>A, NM_000492, Het, LP	PD
CFTR c.579+1G>T, NM_000492, Het, P	PD
CFTR c.772A>G (p.Arg258Gly), NM_000492, Het, LP	PD
CFTR c.772A>G (p.Arg258Gly), NM_000492, Het, LP	PD
CNGB3 c.1148delC (p.Thr383Ilefs*13), NM_019098, Het, P	PD
CNGB3 c.2181_2184delAAAA (p.Glu729Metfs*99), NM_019098, Het, LP	NLoF*
COL7A1 c.3140-2A>G, NM_000094, Het, LP	NLoF
COL7A1 c.6527dup (p.Gly2177Trpfs*113), NM_000094, Het, P	PD
COL7A1 c.7930-1G>C, NM_000094, Het, P	PD
CPT2 c.338C>T (p.Ser113Leu), NM_000098, Het, P	PD
CPT2 c.338C>T (p.Ser113Leu), NM_000098, Het, P	PD
CPT2 c.338C>T (p.Ser113Leu), NM_000098, Het, P	PD
CTNS c.1015G>A (p.Gly339Arg), NM_001031681, Het, P	PD
CYP27A1 c.1016C>T (p.Thr339Met), NM_000784, Het, P	PD
CYP27A1 c.1183C>T (p.Arg395Cys), NM_000784, Het, P	PD
DHCR7 c.1337G>A (p.Arg446Gln), NM_001360, Het, LP	PD
DHCR7 c.1337G>A (p.Arg446Gln), NM_001360, Het, LP	PD
DHCR7 c.278C>T (p.Thr93Met), NM_001360, Het, P	PD
DHCR7 c.452G>A (p.Trp151*), NM_001360, Het, P	PD
DHCR7 c.906C>G (p.Phe302Leu), NM_001360, Het, P	PD
DHCR7 c.964-1G>C (p.?), NM_001360, Het, P	PD
DHCR7 c.964-1G>C (p.?), NM_001360, Het, P	PD
DIS3L2 c.166_167insCACCA (p.Lys56Hisfs*10), NM_152383, Het, LP	NLoF
DIS3L2 c.1835dupC (p.Pro613Alafs*8), NM_152383, Het, LP	NLoF*
DPYD c.1679T>G (p.Ile560Ser), NM_000110, Het, P	PD
DPYD c.1679T>G (p.Ile560Ser), NM_000110, Het, P	PD
DPYD c.1905+1G>A, NM_000110, Het, P	PD
DPYD c.1905+1G>A, NM_000110, Het, P	PD
DPYD c.2846A>T (p.Asp949Val), NM_000110, Het, LP	PD
DPYD c.2846A>T (p.Asp949Val), NM_000110, Het, LP	PD
DPYD c.2846A>T (p.Asp949Val), NM_000110, Het, LP	PD
EYS c.749-1G>C, NM_001142800, Het, LP	PD
EYS c.9102dupT (p.Asn3035*), NM_001142800, Het, LP	NLoF
F11 c.403G>T (p.Glu135*), NM_000128, Het, P	PD
F11 c.595+3A>G, NM_000128, Het, P	PD
FAH c.1021C>T (p.Arg341Trp), NM_000137, Het, No known risk	PD
FAH c.1021C>T (p.Arg341Trp), NM_000137, Het, No known risk	PD
FAH c.1021C>T (p.Arg341Trp), NM_000137, Het, No known risk	PD
FAH c.1021C>T (p.Arg341Trp), NM_000137, Het, No known risk	PD

PRKN c.155delA (p.Asn52Metfs*29), NM_004562, Het, P	PD
PRKN c.823C>T (p.Arg126Trp), NM_004562, Het, P	PD
PRKN c.823C>T (p.Arg275Trp), NM_004562, Het, P	PD
PRKN c.823C>T (p.Arg275Trp), NM_004562, Het, P	PD
PRKN c.823C>T (p.Arg275Trp), NM_004562, Het, P	PD
PYGM c.1094C>T (p.Ala365Val), NM_005609, Het, LP	PD
PYGM c.1094C>T (p.Ala365Val), NM_005609, Het, LP	PD
PYGM c.1094C>T (p.Ala365Val), NM_005609, Het, LP	PD
PYGM c.148C>T (p.Arg50*), NM_005609, Het, P	PD
PYGM c.148C>T (p.Arg50*), NM_005609, Het, P	PD
PYGM c.148C>T (p.Arg50*), NM_005609, Het, P	PD
PYGM c.148C>T (p.Arg50*), NM_005609, Het, P	PD
PYGM c.2262del (p.Lys754Asnfs*49), NM_005609, P	PD
RAD51C c.404G>A (p.Cys135Tyr), NM_058216, Het, LP	PD
RECQL4 c.1223_1224insT (p.Gln408Hisfs*15), NM_004260, Het, LP	PD
RPE65 c.95-2A>T, NM_000329, Het, P	PD
RYR1 c.7615-2A>G, NM_000540, Het, LP	NLoF
SBDS c.183_184delTAinsCT (p.Lys62*), NM_016038, Het, P	PD
SBDS c.183_184delTAinsCT (p.Lys62*), NM_016038, Het, P	PD
SBDS c.183_184delTAinsCT (p.Lys62*), NM_016038, Het, P	PD
SERPINA1 c.187C>T (p.Arg63Cys), NM_001127701, Het, P	PD
SERPINA1 c.187C>T (p.Arg63Cys), NM_001127701, Het, P	PD
SERPINA1 c.187C>T (p.Arg63Cys), NM_001127701, Het, P	PD
SERPINA1 c.863A>T (p.Glu288Val), NM_001127701, Het, No known risk	PD
SERPINA1 c.863A>T (p.Glu288Val), NM_001127701, Het, No known risk	PD
SERPINA1 c.863A>T (p.Glu288Val), NM_001127701, Het, No known risk	PD
SERPINA1 c.863A>T (p.Glu288Val), NM_001127701, Het, No known risk	PD
SERPINA1 c.863A>T (p.Glu288Val), NM_001127701, Het, No known risk	PD
SERPINA1 c.863A>T (p.Glu288Val), NM_001127701, Het, No known risk	PD
SERPINA1 c.863A>T (p.Glu288Val), NM_001127701, Het, No known risk	PD
SERPINA1 c.863A>T (p.Glu288Val), NM_001127701, Hom, No known risk	PD
SERPINA1 c.863A>T (p.Glu288Val), NM_001127701, Hom, No known risk	PD
SERPINA1 c.863A>T (p.Glu288Val), NM_001127701, Hom, No known risk	PD
SERPINA1 c.863A>T (p.Glu288Val), NM_001127701, Hom, No known risk	PD
SERPINA1 Pi*MS, NM_001127701, Het, No known risk	PD
SERPINA1 Pi*MS, NM_001127701, Het, No known risk	PD
SERPINA1 Pi*MS, NM_001127701, Het, No known risk	PD
SERPINA1 Pi*MS, NM_001127701, Het, No known risk	PD
SERPINA1 Pi*MS, NM_001127701, Het, No known risk	PD
SERPINA1 Pi*MS, NM_001127701, Het, No known risk	PD
SERPINA1 Pi*MS, NM_001127701, Het, No known risk	PD
SERPINA1 Pi*MS, NM_001127701, Het, No known risk	PD
SERPINA1 Pi*MS, NM_001127701, Het, No known risk	PD

FAH c.1021C>T (p.Arg341Trp), NM_000137, Het, No known risk	PD
FAH c.1021C>T (p.Arg341Trp), NM_000137, Het, No known risk	PD
FAH c.1021C>T (p.Arg341Trp), NM_000137, Het, No known risk	PD
FAH c.1021C>T (p.Arg341Trp), NM_000137, Het, No known risk	PD
FAH c.1021C>T (p.Arg341Trp), NM_000137, Het, No known risk	PD
FAH c.1021C>T (p.Arg341Trp), NM_000137, Het, No known risk	PD
FAH c.1021C>T (p.Arg341Trp), NM_000137, Het, No known risk	PD
FAH c.1021C>T (p.Arg341Trp), NM_000137, Het, No known risk	PD
FAH c.1021C>T (p.Arg341Trp), NM_000137, Het, No known risk	PD
FAH c.1021C>T (p.Arg341Trp), NM_000137, Het, No known risk	PD
FAH c.1021C>T (p.Arg341Trp), NM_000137, Het, No known risk	PD
FAH c.554-1G>T, NM_000137, Het, P	PD
FAH c.554-1G>T, NM_000137, Het, P	PD
FAM161A c.1355_1356delCA (p.Thr452Serfs*3), NM_001201543, P	PD
FAN1 c.1397_1398delCT (p.Ser466*), NM_014967, Het, LP	NLoF
FAN1 c.2854C>T (p.Arg952*), NM_014967, Het, LP	PD
FANCA c.1115_1118delTTGG (p.Val372Alafs*42), NM_000135, Het, P	PD
FANCA c.2639G>A (p.Arg880Gln), NM_000135, Het, LP	PD
FANCA c.3066+1G>A, NM_000135, Het, LP	PD
FANCA c.3913C>T (p.Leu1305Phe), NM_000135, Het, LP	PD
FANCC c.520C>T (p.Arg174*), NM_000136, Het, LP	PD
FANCE c.929dupC (p.Val311Serfs*2), NM_021922, Het, LP	PD
FANCI c.3041G>A (p.Cys1014Tyr), NM_001113378, Het, LP	PD
FANCL c.1066_1067delAG (p.Ser356Phefs*2), NM_001114636, Het, LP	PD
FKBP14 c.362dupC (p.Glu122Argfs*7), NM_017946, Het, P	PD
G6PC c.808G>C (p.Gly270Arg), NM_000151, Het, LP	PD
GAA c.1798C>T (p.Arg600Cys), NM_000152, Het, P	PD
GAA c.2167G>A (p.Val723Met), NM_000152, Het, LP	PD
GAA c.2237G>C (p.Trp746Ser), NM_000152, Het, LP	PD
GAA c.-32-13T>G, NM_000152, Het, P	PD
GAA c.-32-13T>G, NM_000152, Het, P	PD
GAA c.-32-13T>G, NM_000152, Het, P	PD
GALC c.1630G>A (p.Asp544Asn), NM_000153, Het, LP	PD
GALC c.1896_1900delCACGT (p.Thr633Asnfs*3), NM_000153, Het, LP	PD
GALT c.425T>A (p.Met142Lys), NM_000155, Het, P	PD
GALT c.563A>G (p.Gln188Arg), NM_000155, Het, P	PD
GALT c.-67-52_-67-49delGTCA, NM_000155, Het, ER	PD
GALT c.-67-52_-67-49delGTCA, NM_000155, Het, ER	PD
GALT c.-67-52_-67-49delGTCA, NM_000155, Het, ER	PD
GALT c.-67-52_-67-49delGTCA, NM_000155, Het, ER	PD
GALT c.-67-52_-67-49delGTCA, NM_000155, Het, ER	PD
GALT c.-67-52_-67-49delGTCA, NM_000155, Het, ER	PD
GALT c.-67-52_-67-49delGTCA, NM_000155, Het, ER	PD
GALT c.-67-52_-67-49delGTCA, NM_000155, Het, ER	PD
GALT c.-67-52_-67-49delGTCA, NM_000155, Het, ER	PD
GALT c.-67-52_-67-49delGTCA, NM_000155, Het, ER	PD
GALT c.-67-52_-67-49delGTCA, NM_000155, Het, ER	PD
GALT c.-67-52_-67-49delGTCA, NM_000155, Het, ER	PD
GALT c.-67-52_-67-49delGTCA, NM_000155, Het, ER	PD

SERPINA1 Pi*MS, NM_001127701, Het, No known risk	PD
SERPINA1 Pi*MS, NM_001127701, Het, No known risk	PD
SERPINA1 Pi*MS, NM_001127701, Het, No known risk	PD
SERPINA1 Pi*MS, NM_001127701, Het, No known risk	PD
SERPINA1 Pi*MS, NM_001127701, Het, No known risk	PD
SERPINA1 Pi*MS, NM_001127701, Het, No known risk	PD
SERPINA1 Pi*MS, NM_001127701, Het, No known risk	PD
SERPINA1 Pi*MS, NM_001127701, Het, No known risk	PD
SERPINA1 Pi*MS, NM_001127701, Het, No known risk	PD
SERPINA1 Pi*MS, NM_001127701, Het, No known risk	PD
SERPINA1 Pi*MS, NM_001127701, Het, No known risk	PD
SERPINA1 Pi*MS, NM_001127701, Het, No known risk	PD
SERPINA1 Pi*MS, NM_001127701, Het, No known risk	PD
SERPINA1 Pi*MS, NM_001127701, Het, No known risk	PD
SERPINA1 Pi*MS, NM_001127701, Het, No known risk	PD
SERPINA1 Pi*MS, NM_001127701, Het, No known risk	PD
SERPINA1 Pi*MS, NM_001127701, Het, No known risk	PD
SERPINA1 Pi*MS, NM_001127701, Het, No known risk	PD
SERPINA1 Pi*MS, NM_001127701, Het, No known risk	PD
SERPINA1 Pi*MS, NM_001127701, Het, No known risk	PD
SERPINA1 Pi*MS, NM_001127701, Het, No known risk	PD
SERPINA1 Pi*MS, NM_001127701, Het, No known risk	PD
SERPINA1 Pi*MS, NM_001127701, Het, No known risk	PD
SERPINA1 Pi*MS, NM_001127701, Het, No known risk	PD
SERPINA1 Pi*MS, NM_001127701, Het, No known risk	PD
SERPINA1 Pi*MS, NM_001127701, Het, No known risk	PD
SERPINA1 Pi*MS, NM_001127701, Het, No known risk	PD
SGCB c.325C>T (p.Arg109*), NM_000232, Het, LP	PD
SLC12A3 c.1928C>T (p.Pro643Leu), NM_001126108, Het, P	PD
SLC12A3 c.1930delC (p.Gln644Serfs*28), NM_000339, Het, LP	PD
SLC12A3 c.2221G>A (p.Gly741Arg), NM_000339, Het, P	PD
SLC12A3 c.2864G>A (p.Arg955Gln), NM_001126108, Het, P	PD
SLC12A3 c.438C>A (p.Cys146*), NM_000339, Het, LP	PD
SLC12A6 c.2042+1G>A, NM_133647, Het, LP	NLoF
SLC12A6 c.316+1G>A, NM_133647, Het, LP	PD
SLC17A5 c.918T>G (p.Tyr306*), NM_012434, Het, P	PD
SLC26A2 c.835C>T (p.Arg279Trp), NM_000112, Het, P	PD
SLC26A2 c.835C>T (p.Arg279Trp), NM_000112, Het, P	PD
SLC26A2 c.835C>T (p.Arg279Trp), NM_000112, Het, P	PD
SLC26A4 c.1003T>C (p.Phe335Leu), NM_000441, Het, P	PD
SLC26A4 c.1489G>A (p.Gly497Ser), NM_000441, Het, P	PD
SLC2A10 c.394C>T (p.Arg132Trp), NM_030777, Het, LP	PD
SLX4 c.4259dupC (p.Ile1421Asnfs*4), NM_032444, Het, LP	NLoF*
SPG11 c.1609delA (p.Ile537*), NM_025137, Het, LP	NLoF
SPG11 c.5020delA (p.Arg1674Aspfs*19), NM_025137, Het, LP	NLoF
SPG11 c.6737_6740delTTGA (p.Ile2246Serfs*15), NM_025137, Het, P	PD
SPG11 c.6744delT (p.Gln2249Serfs*1), NM_025137, Het, LP	NLoF
SPG11 c.6832_6833del (p.Ser2278Leufs*61), NM_025137, Het, P	PD
SPG11 c.733_734delAT (p.Met245Valfs*2), NM_025137, Het, P	PD

GALT c.-67-52_-67-49delGTCA, NM_000155, Het, ER	PD
GALT c.-67-52_-67-49delGTCA, NM_000155, Het, ER	PD
GBA c.1226A>G (p.Asn409Ser), NM_001005741, Het, P	PD
GBA c.1229G>C (p.Asp409His), NM_000157, Het, P	PD
GBA c.1259G>A (p.Trp420*), NM_000157, Het, P	PD
GBE1 c.691+2T>C, NM_000158, Het, P	PD
GCDH c.1198G>A (p.Val400Met), NM_000159, Het, P	PD
GCDH c.1198G>A (p.Val400Met), NM_000159, Het, P	PD
GCDH c.1204C>T (p.Arg402Trp), NM_000159, Het, P	PD
GCDH c.680G>C (p.Arg227Pro), NM_000159, Het, P	PD
GCDH c.877G>A (p.Ala293Thr), NM_000159, Het, P	PD
GCDH c.877G>A (p.Ala293Thr), NM_000159, Het, P	PD
GCDH c.877G>A (p.Ala293Thr), NM_000159, Het, P	PD
GJB2 c.101T>C (p.Met34Thr), NM_004004, Het, P	PD
GJB2 c.101T>C (p.Met34Thr), NM_004004, Het, P	PD
GJB2 c.101T>C (p.Met34Thr), NM_004004, Het, P	PD
GJB2 c.101T>C (p.Met34Thr), NM_004004, Het, P	PD
GJB2 c.101T>C (p.Met34Thr), NM_004004, Het, P	PD
TRDN c.1914delA (p.Arg639Glufs*10), NM_006073, Het, LP	NLoF
TSHR c.202C>T (p.Pro68Ser), NM_000369, Het, LP	PD
TSHR c.202C>T (p.Pro68Ser), NM_000369, Het, LP	PD
TYR c.1217C>T (p.Pro406Leu), NM_000372, Het, P	PD
TYR c.1217C>T (p.Pro406Leu), NM_000372, Het, P	PD
TYR c.1217C>T (p.Pro406Leu), NM_000372, Het, P	PD
TYR c.24C>A (p.Cys8*), NM_000372, Het, LP	PD
TYR c.823G>T (p.Val275Phe), NM_000372, Het, P	PD
USH2A c.1214delA (p.Asn405Ilefs*3), NM_206933, Het, P	PD
USH2A c.2276G>T (p.Cys759Phe), NM_206933, Het, P	PD
USH2A c.2276G>T (p.Cys759Phe), NM_206933, Het, P	PD
USH2A c.2299delG (p.Glu767Serfs*21), NM_206933, Het, P	PD
USH2A c.3883C>T (p.Arg1295*), NM_206933, Het, LP	PD
USH2A c.6937G>T (p.Gly2313Cys), NM_206933, Het, LP	PD
USH2A c.9304C>T (p.Gln3102*), NM_206933, Het, LP	PD
WRN c.2855C>A (p.Ser952*), NM_000553, Het, LP	PD
XPC c.1643_1644delTG (p.Val548Alafs*25), NM_004628, Het, P	PD

Supplementary Table S2. Variants detected in genes of autosomal recessive or X-linked inheritance (carrier status). HET: Heterozygous; HOM: Homozygous; ER: Established Risk; P: Pathogenic; LP: Likely Pathogenic; PD: Previously described; NLoF: Novel loss of function; NLoF*: Novel loss of function variant at the time of analysis.

Gene	Cases	Condition	Observed Carrier Frequency in study cohort	Carrier Frequency in literature[a]
CFTR	23	Cystic fibrosis	1/17	1/20
GJB2	23	Non-syndromic hearing loss and deafness	1/17	1/24
ATP7B	14	Wilson disease	1/28	1/39
PAH	9	Phenylketonuria	1/44	1/21
ACADM	8	Medium-chain acyl-coenzyme A dehydrogenase deficiency	1/50	1/57
ALDOB	8	Hereditary fructose intolerance	1/50	1/71
MEFV	8	Familial Mediterranean fever	1/50	1/11
GBA	3	Gaucher disease	1/133	1/18

Supplementary Table S3. Carrier frequency in a subset of genes related to recessive conditions compared to literature. (a)Carrier frequency from ethnicity with highest carrier frequency; does not include Native American, Pacific Islander, Finnish, or Unknown ethnicity (Taber et al., 2022).

Gene	Patient	Drug	Medical area
CYP2C19	125	Clopidogrel	Haematology, cardiovascular
TPMT	3	Azatioprine, Mercaptopurine, Thioguanine	Transplant medicine, infectious diseases and immunological disorders, oncology
DPYD	6	Capecitabine, Fluorouracil, Tegafur	Oncology
G6PD	3	Dapsone	Infectious diseases and immunological disorders
CYP2D6	5	Codeine, Tramadol	Pain medicine

Supplementary Table S4. Genes in which a serious drug interaction was found, and the drugs and medical area affected.
